# Perceptions of Illness Control, Coherence, and Self-Efficacy Following a Web-Based Lifestyle Program for Multiple Sclerosis: A Qualitative Analysis of Semistructured Interviews

**DOI:** 10.2196/60240

**Published:** 2024-11-29

**Authors:** Rebekah Davenport, William Bevens, Sandra Neate, Pia Jelinek, Maggie Yu, George Jelinek, Jeanette Reece

**Affiliations:** 1 Melbourne School of Psychological Sciences Faculty of Medicine, Dentistry & Health Sciences The University of Melbourne Melbourne Australia; 2 Department of Psychiatry IN STEP Children’s Mental Health Research Center University of California, San Diego La Jolla, CA United States; 3 Melbourne School of Theology Centre for Epidemiology and Biostatistics The University of Melbourne Melbourne Australia

**Keywords:** illness perceptions, personal control perceptions, illness coherence, self-efficacy, lifestyle modification, disease management, multiple sclerosis, MS, qualitative, health behavior, thematic analysis

## Abstract

**Background:**

Evidence suggests that illness perceptions held by people living with multiple sclerosis (MS) impact affective distress and physical health outcomes. In a randomized controlled trial, we developed 2 MS Online Courses—the standard care course and the intervention course (IC). The IC was adapted from an evidence-based lifestyle program. Modifying lifestyle risk factors offers an opportunity to impact illness perceptions. Research on illness perceptions in people living with MS has focused predominately on quantitative methods.

**Objective:**

This study aimed to explore the experiences and health outcomes of randomized controlled trial participants, including individuals’ perceived changes in attitudes toward MS and their health (ie, illness perceptions).

**Methods:**

Qualitative responses provided by 38 participants (22 in the IC and 16 in the standard care course) were derived from semistructured interviews 1 month after completing the MS Online Course. The impact of course engagement and lifestyle modification on illness perceptions was explored using inductive thematic analysis.

**Results:**

Themes of changes in illness perceptions were most prominent in the IC arm. Qualitative responses from 22 people living with MS informed the development of three themes: “self-efficacy for disease management,” “personal control,” and “illness coherence.”

**Conclusions:**

Findings provide novel insights into the formation and modification of illness perceptions. Lifestyle modification may serve as a valuable adjunct to clinician-administered therapies, improving the sense of personal control over MS, illness coherence, and self-efficacy for disease management.

**Trial Registration:**

Australian New Zealand Clinical Trials Registry ACTRN12621001605886; https://tinyurl.com/2vyve9p9

**International Registered Report Identifier (IRRID):**

RR2-10.1186/s12883-023-03298-0

## Introduction

People living with multiple sclerosis (MS) are tasked with adjusting to substantial prognostic uncertainty, in the absence of clarity regarding the etiology of symptoms and without access to a cure. Critical events, such as receiving an MS diagnosis and ongoing illness stressors, can disrupt emotional equilibrium [1]. It is well established that depressive, anxiety, and stress symptoms are highly prevalent in the population with MS [2]. Approximately 30% of people living with MS report clinically significant depressive symptoms, while 34% experience symptoms of anxiety [2]. Seminal works by Lazarus and Folkman [3] and Lazarus and Opton [4] on cognitive appraisal propose that the psychological impact of an event (eg, diagnosis and relapse) is partially impacted by an individual’s appraisal of the event and their self-efficacy to access and use effective coping responses. Leventhal [5] and Leventhal et al [6] developed the Common-Sense Model of Self-Regulation theory (CSM) to formulate how attitudes and beliefs are specifically formed in response to chronic illness and health events. Research has examined the impact of illness perceptions in people living with MS, highlighting significant contributions of perceptions relating to perceived personal control over MS, a strong illness identity (attribution of physical symptoms to disease), and illness coherence (perceived understanding of MS) on depressive symptoms [7]. Furthermore, self-efficacy perceptions have been shown to impact symptom severity and disability [8] and quality of life (QoL) [9].

Identifying factors predictive of health outcomes and amenable to intervention is critical for fostering hope in disease management, particularly in the context of a highly uncertain disease such as MS. Given their robust links to mental and physical health outcomes in people living with MS, illness perceptions hold substantial clinical significance. In addition, interventional data demonstrate the amenability of illness perceptions. Changes to illness perceptions, facilitated by cognitive behavioral therapy, have been shown to coincide with decreases in depressive and anxiety symptoms [10].

Involving health care practitioners, including mental health professionals, in MS care is crucial but often regarded as time-intensive, difficult to access, and potentially costly [11]. As such, there has been an emerging focus on lifestyle self-management as an intervention for people living with MS, supplemental to clinician-administered psychological treatments and pharmaceutical disease-modifying therapies. Emerging evidence supports the effectiveness of lifestyle self-management interventions, encompassing improved diet quality, increased exercise, mindfulness practices, and stress management, in enhancing QoL [12,13] and improving depressive and anxiety symptoms [14]. Furthermore, self-management interventions place the patient at the center of their own care, emphasizing their responsibility and control over identifying and addressing health challenges.

According to the CSM for chronic disease populations [5,6], exposure to new and alternative information from expert sources is important in influencing the nature and severity of illness perceptions. When presented within the context of a programmatic, educational intervention delivered by experts, lifestyle modification information may provide people living with MS with the required scaffolding to reevaluate their mental representations of illness and efficacy to manage their own MS. To date, research into illness perceptions in the population living with MS has almost exclusively been conducted within a quantitative research paradigm. While findings offer important insights into group-level relationships between illness perceptions and health outcomes in MS, they are unable to address questions relating to the highly-varied individual experience of MS or the impact of self-managed lifestyle modifications on illness perceptions. Few studies have explored the patient perspective on MS using qualitative methods [15]. This information holds considerable value for medical and allied health professionals in tailoring treatment plans for patients with MS [14]. Providing additional options for multimodal lifestyle interventions may enrich the overall approach to patient care.

This study is an additional qualitative analysis to the larger ancillary randomized controlled trial (RCT) that assessed the effectiveness of the MS Online Course (MSOC) [16]. The MSOC provided information tailored on lifestyle-related risk factors for people living with MS, compared with a standard care course (SCC) with general lifestyle information. This qualitative study aimed to assess individuals’ perceived changes in attitudes toward MS and health (ie, illness perceptions) 1 month after completing the MSOC. Responses were obtained through semistructured interviews and analyzed using inductive thematic analysis.

## Methods

### Ethical Considerations

The study involving humans was conducted according to the guidelines of the Declaration of Helsinki and approved by The University of Melbourne Human Research Ethics subcommittee (ID: 22140). This study received approval from the University of Melbourne Human Research Ethics subcommittee on November 2, 2021 (ID: 1851781.2). This ancillary RCT is a CONSORT-R (Consolidated Standards of Reporting Trials–Routine)–compliant RCT (Multimedia Appendix 1). The RCT protocol was reviewed and approved by the Australian New Zealand Clinical Registry on November 25, 2021 (ACTRN12621001605886). Written informed consent was obtained from all participants before inclusion in the RCT with additional verbal consent obtained from all participants in this qualitative study to undertake semistructured qualitative interviews for the purposes of this research. This qualitative study adheres to the Consolidated Criteria for Reporting Qualitative Research (COREQ) [17] and the American Psychological Association (APA) Style Journal Article Reporting Standards [18]. Participants received no compensation for their involvement. Qualitative data are presented anonymously, with participants categorized by study arm and assigned random numbers (1-16 for the SCC; 1-22 for the intervention course [IC]).

### Study Design and Procedure

The MSOC effectiveness RCT is a randomized, single-blinded trial of a 6-week online lifestyle modification intervention that follows participants who completed the MSOC over a 2.5-year period. Consequently, short-term primary RCT outcomes were not yet available at the execution of this study.

Details of the MSOC have been comprehensively described previously [16]. Participants were recruited internationally through MS society websites (eg, MS Australia), research newsletters (eg, Clinical Trials Australia), Instagram (Meta), and public MS Facebook (Meta) groups focused on the dissemination of health information. Eligible participants were aged 18 years or older, fluent in English, and self-reported a physician’s diagnosis of MS. Exclusion criteria included significant comorbid neurological conditions (eg, stroke) or chronic disease and participation in another RCT. At baseline (0 months), participants completed an online survey within the first MSOC education module, covering sociodemographic and disease variables (eg, MS subtype), lifestyle habits, and physical and mental health outcomes.

The MSOC encompassed 2 courses (study arms)—the IC and the SCC—developed by researchers in collaboration with people living with MS to address lifestyle-related risk factors. The objective is to compare the effectiveness of each course in a larger RCT for improving QoL and health outcomes. Both the IC and SCC consist of seven modules: (1) Introduction, (2) Eat Well, (3) Sunlight and Vitamin D, (4) Exercise, (5) Meditation and Mind-body Connection, (6) Medication and Family Prevention, and (7) Conclusion. The SCC provided general lifestyle information from reputable MS websites, while the IC delivered information tailored to people living with MS, based on an evidence-based MS program [19] (Multimedia Appendix 2).

### Qualitative Study

#### Participant Recruitment

Participants of the RCT who completed all course modules, the precourse baseline survey, and postcourse evaluation survey across both study arms (n=53) were invited by email to participate in a 45-60 minute semistructured interview 1 month after completing the course (Multimedia Appendix 3). In total, 38 participants accepted the invitation. Written informed consent was obtained from all participants in the baseline survey, within the first MSOC module of the RCT, and verbal consent was obtained before participating in semistructured interviews purposed for qualitative analyses.

Demographic and clinical quantitative data presented in the current study were obtained from the pre-course baseline survey. The Hospital Anxiety and Depression Scale (HADS) [20] was reported to characterize the severity of self-reported depressive and anxiety symptoms. The HADS, used extensively in the population living with MS (eg, Dahl et al [21] and Janssens et al [22]), has demonstrated accurate sensitivity and specificity for major depressive disorder (90% and 87%, respectively) and generalized anxiety disorder (88% and 81%, respectively) within the population living with MS [23]. Ambulation levels were evaluated using the Patient-Determined Disease Steps (PDDS) scale [24], recognized as a guide of overall disability in individuals with mild-to-moderate disabilities due to MS [25].

#### Interview Procedure

Participants were invited by email to participate in semistructured interviews 1 month after completing the MSOC. Interviews occurred at a time convenient for participants between October 14, 2022, and November 16, 2022, and were conducted by SN (lead interviewer), RD, JR, and PJ using Zoom (Zoom Video Communications, Inc) software licensed by The University of Melbourne or WhatsApp (Meta). Interviewers had a research background and either (1) had substantial experience conducting qualitative research or (2) were highly trained health care professionals routinely involved in the clinical management of MS and other chronic diseases. There were minor variations in the interview scripts for the intervention versus the standard care group. Interview questions explored perceived changes in attitudes toward MS and health, initial health changes, and broader aspects of the course experience (eg, engagement with the online forum and views of course content; Multimedia Appendix 4).

To ensure consistency in the delivery of the interview schedule between interviewers, authors (RD, JR, and PJ) observed 1 interview conducted by the lead interviewer (SJ). Minor changes to the structure of the interview schedule were made following the 3 initial interviews with the intention of ensuring the schedule followed a coherent, logical order (eg, all questions probing mental health variables were asked consecutively). With participant permission, interviews were audio recorded and transcribed verbatim, using the voice recognition software Temi (Rev) [26]. Transcripts were saved in a restricted folder and were accessed by interviewers. Transcript text was altered for purposes such as upholding participant confidentiality by redacting sensitive disclosures as requested by participants and ensuring that text were a verbatim account participants’ verbal responses. Transcripts were imported into NVivo qualitative data analysis software (version 12, 2018; Lumivero [formerly QSR International]). In line with best-practice data management, document coding and theme development were maintained within NVivo outputs.

#### Thematic Analysis

Inductive, reflexive thematic analysis was used as a means of identifying, analyzing, and reporting patterns in the data [27]. Reflexive thematic analysis was selected as it allows for researcher autonomy in the processes of determining themes (latent or semantic) and establishing the most relevant analysis to use (inductive vs theoretical). This study applied the 6 distinct phases of thematic analysis: data familiarization, generating codes, constructing themes, reviewing potential themes, defining and naming themes, and producing the report [28]. The dataset was divided into 2, according to the IC and SCC arms, and both were analyzed and coded independently by RD and WB, respectively.

Following data familiarization and coding, the research team met to review potential themes to ensure alignment with the raw data and participants’ perspectives. To enhance transparency, verbatim quotes have been included to illustrate themes, allowing readers to assess the validity of researchers’ interpretations.

**Figure 1:**
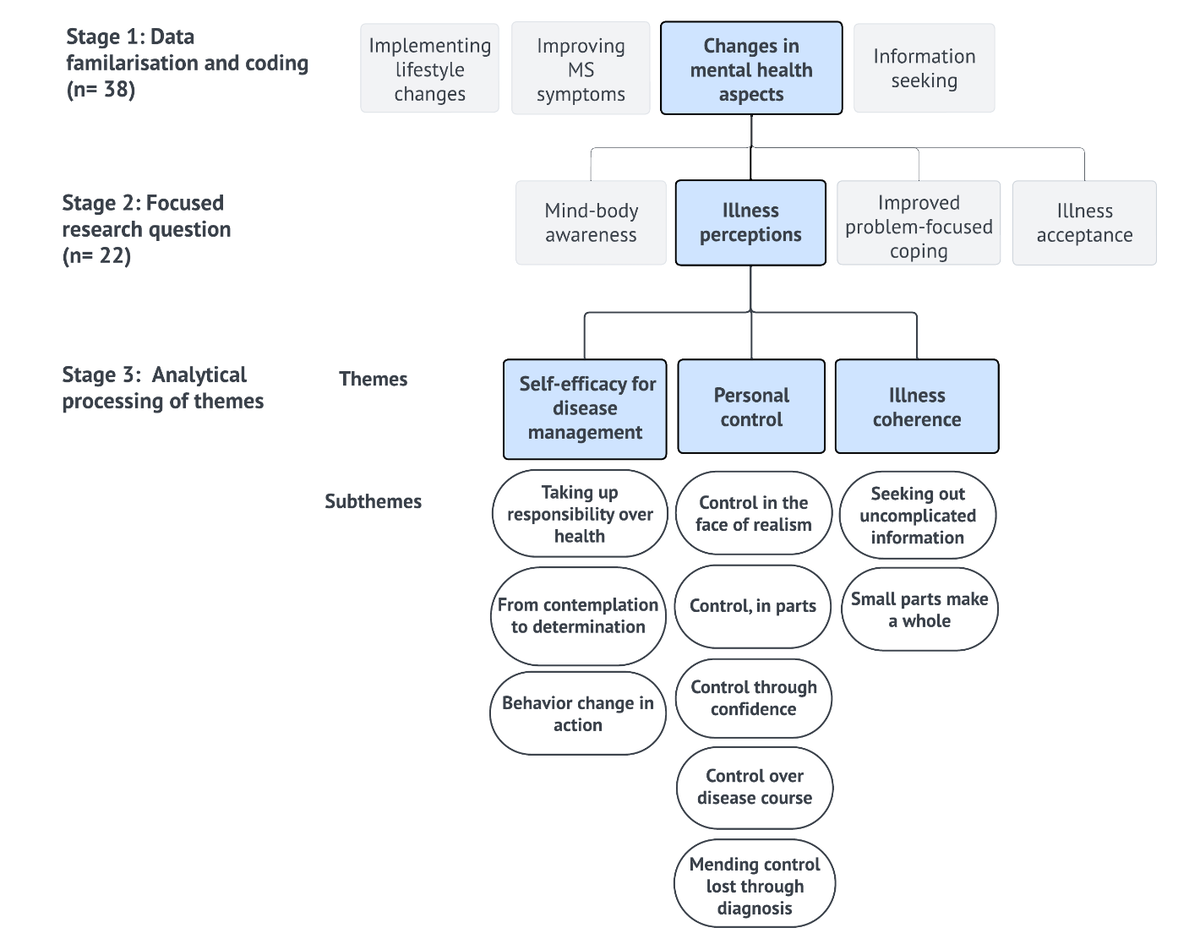
Schematic of the qualitative analysis process stages 1-3. MS: multiple sclerosis.

Participants in the SCC arm reported some changes in mental health; however, these were varied and often attributed to external factors. As such, only data from participants in the IC arm were retained. In total, 4 superordinate themes were discerned from the data corpus (Figure 1). Due to their distinct nature and relevance to diverse fields, the research team partitioned the data based on these superordinate themes and conducted separate analyses. This study focuses on the superordinate theme “changes in mental health aspects,” delving into psychological phenomena such as participants’ experiences in modifying illness perceptions over the course. Another superordinate theme, “information seeking,” which addresses online information-seeking behaviors among people living with MS, was reported separately [29]. The remaining 2 themes required quantitative examination to derive their meaning, in alignment with the larger RCT’s goals of quantifying changes in MS symptoms and lifestyle after MSOC completion.

## Results

### Participant Characteristics

Table 1 provides demographic and clinical characteristics. Among the 22 participants who completed the IC (mean age 48, SD 12 years), most identified as female (19/22, 86%) and self-reported a relapsing-remitting MS phenotype (18/22, 82%). Most participants scored within the “normal” (<8 HADS score) ranges for depression (19/22, 86%), while over half reported “normal” anxiety levels (13/22, 59%). Clinically significant fatigue was reported by a large proportion (13/22, 59%), and 68% (15/22) of participants had no or mild disability.

**Table 1:** Self-reported demographic and clinical characteristics of the multiple sclerosis online course interventional arm interviewees (n=22) at baseline.

Demographics			Statistical value
**Age (years), n (%)**			48.14 (12.25)
**Gender, n (%)**			
	Men		3 (14)
	Women		19 (86)
	Other		0 (0)
**Country of residence, n (%)**			
	Australia or New Zealand		5 (23)
	United States or Canada		5 (23)
	United Kingdom		3 (14)
	Other		9 (41)
**Highest educational level, n (%)**			
	Postgraduate degree		7 (32)
	University degree		7 (32)
	Secondary education		2 (9)
	Higher education qualification (eg, certificate and diploma)		6 (27)
**Employment status, n (%)**			
	Currently employed		13 (59)
	Unemployed		8 (36)
	Missing		1 (5)
**Currently in a relationship, n (%)**			
	Yes		14 (64)
	No		8 (36)
**Clinical characteristics**			
	**MS^a^ phenotype, n (%)**		
		RRMS^b^	18 (82)
		PPMS^c^	2 (9)
		SPMS^d^	1 (5)
		Unsure or other	1 (5)
	**Years since MS diagnosis, mean (SD)**		5.36 (4.30)
	**Disability level, mean (SD)**		1.55 (1.82)
		Normal or mild (PDDS^e^≤2), n (%)	15 (68)
		Moderate (PDDS≥3 to 5), n (%)	6 (27)
		Severe (PDDS≥6), n (%)	1 (5)
	**DMT^f^ use, n (%)**		
		Yes	13 (59)
		No	9 (41)
	**Comorbidities, n (%)**		
		Yes	16 (73)
		No	4 (18)
		Missing	2 (9)
		Anemia or blood disease	1 (5)
		Anxiety	4 (18)
		Back pain	4 (18)
		Lung disease	2 (9)
		Other (eg, high cholesterol)	11 (50)
	**Fatigue levels, mean (SD)**		4.81 (1.59)
		Normal or mild, n (%)	9 (41)
		Clinically significant (FSS^g^>5), n (%)	13 (59)
	**Depressive symptoms, mean (SD)**		4.91 (3.07)
		Normal (HADS^h^<8), n (%)	19 (86)
		Mild-moderate (HADS≥8 to 10), n (%)	1 (5)
		Clinically significant (HADS≥11), n (%)	2 (9)
	**Anxiety symptoms, mean (SD)**		7.14 (4.61)
		Normal (HADS<8), n (%)	13 (59)
		Mild-moderate (HADS≥8 to 10), n (%)	5 (23)
		Clinically significant (HADS≥11), n (%)	4 (18)

^a^MS: multiple sclerosis.

^b^RRMS: relapsing-remitting multiple sclerosis.

^c^PPMS: primary progressive multiple sclerosis.

^d^SPMS: secondary progressive multiple sclerosis.

^e^PDDS: Patient-Determined Disease Steps.

^f^DMT: disease-modifying treatments.

^g^FSS: fatigue severity scale.

^h^HADS: Hospital Anxiety and Depression Scale.

### Themes

#### Overview

Within “changes in mental-health aspects,” 3 themes relating to illness perceptions were identified. The first theme, “self-efficacy for disease management,” explored perceptions of increased confidence and motivation to manage MS, gained through engaging with and applying lifestyle modification information. Data relating to this theme reflected beliefs about one’s capacity to execute behaviors necessary for disease management, resembling the concept of self-efficacy assessed in the Multiple Sclerosis Self-Efficacy (MSSE) Scale [30], the gold-standard assessment for self-efficacy in persons with MS. Thus, the designation of this theme title was deemed appropriate.

The second theme, “personal control,” explored perceptions of improved sense of personal control over MS, developed through engaging with and applying lifestyle modification information from the IC. Finally, the third theme, “illness coherence,” explored individuals’ experiences of an increased and more coherent cognitive representation of their MS. These 2 themes were named due to the high similarity to the factors “personal control” and “illness coherence,” as described by the Illness Perceptions Questionnaire (IPQ) [31].

Quotations are reported to illustrate themes, identified by the study arm (IC) and participant number.

#### Self-Efficacy for Disease Management

The theme “self-efficacy for disease management” explored individuals’ perceptions of increased confidence and motivation gained through undertaking the IC, and by commencing the recommended lifestyle changes provided in the IC. Increased self-efficacy had 3 effects, referred to as subthemes, including “taking responsibility over health,” “from contemplation to determination,” and “behavior change in action.”

Participants described improvements in their self-efficacy to manage MS through learning about lifestyle modifications for diet, exercise, and mindfulness practice. Course information appeared to foster motivation to adopt a future-oriented focus and prioritize what changes may be important for improving health, based on evidence presented in the course. Equipped with this lifestyle-modification information, participants described feeling an increased sense of confidence to take up responsibility over their own health and motivation to make recommended lifestyle changes.

I felt confident (from the course) that I now have the data behind making changes. I can believe it.

IC18

It’s had a positive effect because I feel more confidence that I know what I need to do and that I’ll be able to do it.

IC6

Lifestyle modification information increased participants’ sense of responsibility and ownership over managing their health, enhancing their confidence to adopt course recommendations and seek further lifestyle-related information.

The course gave me a launching pad of things I can look at myself.

IC14

Having a course like this has helped me know that I am on the right path. I’m doing the right things. I’ve got more information while I was doing the course that boosted me.

IC9

Increased confidence and motivation, stemming from engagement, enabled many participants to progress through stages of change in lifestyle management, as delineated by the Stages of Change Model [32]. Improvements in self-efficacy facilitated a shift away from merely contemplating change toward a more determined and committed approach, evident in the development of action plans and intentions to make life-overhauling changes.

Now I know what I need to do, to achieve what I want to achieve. I know how to get there. I’ve got a plan.

IC6

Some set specific targets for change, such as supplementing with omega-3 fatty acids, increasing vitamin-D exposure, exercising, and re-engaging with mindfulness techniques including mindful breathing and awareness. For some, this included introducing the first form of intentional exercise in years.

It helped. I already knew some of the changes I needed to make but it pushed me into making them. So, you know, in addition to just relying on vitamin D supplements, I need to actually get out in the sun.

IC22

I have a plan moving forward. I’m really gonna step that (exercise) up. I’ve got a stronger awareness of the boxes I need to tick. I’ve got a target.

IC6

For some, plans had begun to take effect; actions were underway and new patterns of behavior were beginning to form. Participants described improvements in mood and attributed this to the integration of lifestyle changes.

I’m not as depressed as I used to be.

IC2

I’m happier now.

IC20

I feel more in control. Because when you feel that something is stressful if I go and meditate, afterwards I am more relaxed and those bad feelings are better.

IC20

Participant’s responses also raised the possibility of a bidirectional relationship between self-efficacy and change; self-efficacy prompting lifestyle modifications, which may in turn create positive mastery experiences that further enhance self-efficacy.

When I started to do some changing, I started to feel more confident, and overall, it’s been better for my mental-health.

IC18

I definitely feel more confident. It helped me be free of confusion about how my immune system works on MS drugs and leave behind fear of the unknown. I’m remembering to breathe and reduce stress and that it’s important to fuel your body, make good choices, one at a time.

IC4

#### Personal Control

The theme “personal control” explored individuals’ awareness of their improved personal control over their MS through engaging with lifestyle-related modification information. This theme included 5 subthemes relating to different forms of control, including “control in the face of realism,” “control, in parts,” “control through confidence,” “control over disease course,” and “mending control lost through diagnosis.”

Overwhelmingly, participants expressed an increased sense of control over their MS, although tempered by the realism of the disease. They found a balance between focusing on controllable aspects of MS and accepting that their actions alone may not cure their disease.

I believe it [lifestyle modification] won’t necessarily get rid of the MS, but it will improve my day to day, symptom-wise.

IC1

I’m trying to cover all bases. Trying to take some control of a condition that I don’t really have much control of.

IC19

Having accepted that MS is a chronic disease, some participants began to focus on the possibility that lifestyle modifications may help to control the progression of symptoms.

So it (the course), made me realise I can actually, through diet and lifestyle change, I potentially hold off anything getting worse.

IC14

I’m interested in all the factors I can change that are in my hands, to change the course of the disease.

IC12

Many expressed a conservative view of lifestyle modification, stating that even if modifications may not control the disease or symptoms directly, lifestyle modification provided the opportunity to experience a sense of control.

I cannot control it (the disease), but at least I have a feeling I’m controlling it. Which is also very important. It motivated me more to do it (make lifestyle modifications).

IC17

When I was diagnosed I asked my neurologist about the diet changes and she said, ‘as a doctor, I cannot tell you that diet change will help you, but I think you should change your diet because it will make you feel like you’re in control.’ Even if it’s just that this course gave me information on flax-seed oil. It’s something new that gives me an idea that I am at least a bit more in control than I was.

IC17

Participants were prompted to evaluate the utility of all-or-nothing thinking in relation to personal control, that is, the polarity of being “in control” of their MS versus being “out of control” of their MS. Through learning about controllable aspects of health (eg, diet and exercise), participants were able to reconceptualize control as a feeling which varies along a continuum between these 2 possibilities. That is, self-management lifestyle modification offers individuals the opportunity to gain more control over their MS.

I don’t feel like I can control whether or not I’m going to get new lesions…but I’m doing as much as I can within my control, which is to exercise more, continue to eat a health diet, meditation, yoga.

IC4

It (the course) gave me the push to take more control over my life, with my knowledge about lifestyle modification. I think it helped more than I realised at the time.

IC13

The IC made participants feel they could extend their sense of control over their MS and explore other avenues to gain further control of their MS in the future.

It (the course) just made me think ‘...well, wait a minute, I know I can manage this. I know I can control this, or control elements of it.’ The course gave me a bit more motivation to consider, what else can I do here?

IC13

For some, confidence was highly interrelated with control, such that increases in confidence coincided with increases in perceptions of personal control.

I think it helped me. I was sort of 80% there, but after the course, I’m at 100%. It comes hand in hand with more confidence, you feel like you do have more control. I know now what I need to do and it’s only up to me to do it.

IC6

I am not at the stage where I can defend and justify following the diet, but I’m trying my best to get to that stage… confidence for me comes from having a better understanding of the oils and that. I’m not there yet, but I am more confident, and my confidence comes from feeling a little bit of control.

IC19

For 1 newly diagnosed participant, there was a gap between their current reality and a state of perceived control over their MS. However, the IC, and possibly other educational courses, appeared to help them feel more in control.

I completely panicked after I was diagnosed and I was planning things in advance, and I was like, no, I can’t do that because maybe I won’t be able to walk by then…I felt completely out of control the entire time…Once your realise you can’t actually live like that, then that (personal control) slowly comes back and it’s still coming back and this course has helped.

IC15

#### Illness Coherence

The theme of “illness coherence” encompassed individuals’ beliefs in a more coherent understanding of MS and included 2 subthemes: “seeking out uncomplicated information” and “small parts make a whole.”

Participants emphasized the challenges associated with complex health information, complicated by medical terminology, and the barrier this imposes for constructing a coherent representation of MS. In contrast, the IC was regarded as a helpful source of information, scaffolding participants’ understanding of health information.

Medical information is difficult to understand, but the more I read about it, the easier it becomes.

IC1

One participant described the protective effects of uncomplicated communication within the acute adjustment period following diagnosis.

It (the course) has been helpful. Normalising MS, especially for people in their twenties or thirties who are unexpectedly catapulted into a chronic situation. It’s a lot to take in because we’re at a point in our lives where everything feels like it's starting. Then it feels like you’re figuring out a whole new identity. So, simple communication on health information is helpful. This is a lifestyle change, and also the way we view it, or the way we can support others in understanding it is helpful.

IC5

The opportunity to understand and piece together complex health information enabled participants to develop a more coherent mental representation of their disease, which they viewed as being important for their general well-being.

Overall I think it (doing the IC) was good for my wellbeing…more information makes me more informed and then the changes that I’m implementing should be positive on my overall wellbeing.

IC1

This allowed some to begin translating health information into everyday well-being practices, such as mediation, with beneficial results.

I think things have gotten better since the course, I can say that. And the course helped me to put things together, especially for me, meditation, I’m doing a bit more meditation, which I find really does help.

IC9

## Discussion

### Principal Findings

#### Overview

This study assessed participants’ responses 1 month after completing the IC of an online, tailored education lifestyle intervention for people living with MS. To the authors’ knowledge, this study is the first to qualitatively explore the impact of lifestyle modification on illness perceptions in a population living with MS. Specifically, it aimed to understand the sequelae of the IC on individuals’ cognitive representations of their MS. Within the superordinate theme of “changes in mental health aspects,” 3 subthemes were identified—“self-efficacy for disease management,” “personal control” perceptions, and “illness coherence” perceptions. Findings contribute to an evidence base on the role of illness perceptions in understanding the experiences of people living with MS. In addition, findings offer preliminary support for the CSM by demonstrating a pathway between exposure to new and valid information and changes in control perceptions, illness coherence, and self-efficacy beliefs [15].

The positive effects of self-managed lifestyle modification on mental health outcomes, such as depression and anxiety, in the population with MS are well established [13]. This study provides evidence that exposure to lifestyle-related information may yield benefits for individuals’ perceptions of their disease. Illness perceptions have been proposed as cognitive mechanisms underlying changes in depression and anxiety [31] and may be helpful in linking lifestyle modification to positive mental health outcomes. Positioned as a valuable adjunct to clinician-administered therapies, lifestyle modification has the potential to empower individuals to assume greater responsibility over their disease management, thereby enhancing the comprehensiveness of the treatment approach.

#### Deciphering the Connection: Lifestyle Modification and Personal Control

Gaining a sense of control, which is commonly reduced following MS diagnosis [33], was a primary motivating factor for engaging with the MSOC, as identified in an earlier qualitative study nested within a pilot feasibility study of the MSOC [34]. Furthermore, evidence substantiates the importance of personal control perceptions for mental health outcomes in people living with MS (eg, Brown et al [35] and Jopson and Moss-Morris [36]). While the current analysis confirms the importance of personal control perceptions and the impact of the IC on this aspect, it also offers novel insights into the multidimensional nature of personal control perceptions in MS. Findings illustrate how personal control may be formed, the extent of control gained, and its relevance across MS-related experiences.

Participants’ reports suggested a link between control perceptions and confidence, with each potentially influencing the other. Gaining more information about lifestyle modification, helped to bolster individuals’ confidence and enabled them to feel more in control of MS. Importantly, this was tendered by a pragmatic, continuum-based view of control over MS. While some participants described retrospective feelings of helplessness and a lack of control at the time of diagnosis, they acknowledged the possibility of exerting partial control over MS, aided through their engagement with the IC. This is unexpected as most study participants were within the first 5 years of diagnosis, a period marked by increased distress and tendencies toward all-or-nothing cognitive patterns [37]. Notably, most participants in this analysis scored within the normal ranges for depression and anxiety, contrasting findings from previous observational research [2]. This suggests that the current sample may represent a subgroup of people living with MS who have more positive mental health and may face fewer barriers to lifestyle modification. Considering that motivation difficulties and fatigue—hallmark symptoms of depression [38,39]—are known barriers to lifestyle modification [40], future research should explore how mental health influences engagement with lifestyle modification information, the initiation of lifestyle changes, and the extent to which mental health may impact the effectiveness of self-management interventions on personal control perceptions.

#### Building Illness Coherence: When Information Becomes Clear

Participants’ responses in the current study highlighted the changing nature of illness coherence perceptions, triggered by an MS diagnosis, and shaped through their interaction with the IC. One participant described their experience of diagnosis as a sudden shift, “catapulting” them into a situation that required them to reconstruct their identity in midlife. Notably, given that MS is frequently misunderstood and concealed, the period following diagnosis often requires individuals to assert their understanding of MS and how MS uniquely manifests within them [41]. Therefore, sourcing health information represents a high priority for people living with MS [41,42].

The concept of illness coherence represents a relatively new idea, distinct from the construct of “sense of coherence,” which enables individuals to cope with adverse experiences and is regarded as a dispositional trait (ie, a characteristic embedded within the personality) [42]. Sense of coherence has demonstrated predictive power across a spectrum of physical and mental health outcomes in people living with MS [43,44]; however, given its supposed trait-like structure, its amenability to intervention may be limited. In contrast, illness coherence perceptions have been shown to exhibit temporal variability [45], and their connections to mental health outcomes, encompassing depression and anxiety, have been documented in a handful of MS studies (eg, Kroencke et al [46] and Lynch et al [47]) and are firmly established within the context of other chronic disease populations [48].

The CSM posits that exposure to new and expert information may alter illness perceptions, including psychological adjustment outcomes [15]. The present study extends this concept, suggesting a precondition for such change—health information must be presented in a way that can be easily understood by individuals without specialized medical knowledge. Participant responses suggested that their engagement with uncomplicated health information within the IC facilitated a process of reintegration, helping them to “put things together” again, toward a coherent mental representation of MS. Consequently, participants described improvements in well-being and the practical application of information provided in the IC into daily life, including mindfulness.

#### The Role of Self-Efficacy in Advancing Through Stages of Change

Individuals reported increased self-efficacy for disease management following engagement with the IC which manifested in diverse ways. The Stages of Change Model [32] is a highly influential framework for understanding health-related behavior, and its application can lend meaning to the present findings. Through this lens, participant responses can be allocated to 2 different stages of readiness. The first set of responses suggested that the IC helped them overcome ambivalence toward change, progressing from Prochaska and Velicer’s [32] “contemplation stage to preparation or determination” [32]*.* At this stage, individuals had established the belief that lifestyle modification may lead to a healthier life and described planning steps toward change, with some already experiencing positive effects.

The second set of responses suggested a more advanced stage of change, narrating experiences consistent with a transition from “preparation/determination” into “action.” By incorporating recommended adjustments, including increasing exercise, mindfulness practices, and making dietary modifications, participants noted improvements in their well-being, within a mere month, further enhancing their self-efficacy. These responses raise the possibility of a bidirectional relationship between self-efficacy and lifestyle modification changes, whereby changes in 1 factor feed into changes in the other. The pathway from self-efficacy to behavior change has been tested and substantiated in previous research [49], although operating under the assumption that changes in self-efficacy precede behavioral changes.

It should be noted that an individual’s baseline stage of change (ie, where individuals are at in terms of contemplating or actioning change) may predict the likelihood of behavior change [50]. The act of enrolling in and completing a self-guided lifestyle modification course likely signifies contemplation of change. Future research may consider assessing individuals’ baseline self-efficacy for disease management as one index of readiness for change. Tailoring lifestyle modification educational programs according to the individual’s stage of readiness may help to optimize lifestyle modification change. This approach may also help cater to those who are less inclined to engage with lifestyle modification (eg, in the precontemplation stage), as well as those focused on maintaining changes and preventing regression to earlier stages.

### Limitations and Future Research

A 1-month time lag between completion of the MSOC and the interviews yielded insights into the immediate impacts of engagement and implementation of lifestyle modifications. Nonetheless, the long-term effects of the MSOC, specifically the IC, could not be determined due to the scope of this study. Future research should examine changes in illness perceptions to determine if changes are maintained or bolstered through longer-term implementation of lifestyle changes, as individuals witness the benefits of modifications. Of equal importance, researchers should seek to assess cases where individuals initially implemented learned information but experienced a subsequent decline in their efforts over time.

Despite the strengths in the study’s international scope and voluntary sampling, certain participant characteristics were underrepresented. In alignment with the study’s objective to evaluate the course’s impact, a deliberate selection process was necessary wherein only a small percentage of participants who had completed the larger RCT were eligible for qualitative interviews. However, this approach introduces constraints on the generalizability of findings, warranting caution in extending these results to the broader population with MS. Underrepresented characteristics encompass male individuals, individuals from non-WEIRD (Western, educated, industrialized, rich, and democratic) countries, those identifying outside the binary categories of male and female, individuals with progressive MS phenotypes, and those exhibiting moderate-to-higher levels of depressive and anxiety symptoms.

### Conclusions

This study provides evidence for the therapeutic impact of engagement with educational information on lifestyle modification. Representing one of the first qualitative examinations in this area, this study provides insights into the formation and modification of illness perceptions which are commonly linked to mental and physical health outcomes. Findings suggest that the IC, a self-guided online lifestyle modification program, may serve as a valuable adjunct to clinician-administered therapies, improving sense of personal control over MS, disease coherence, and self-efficacy for disease management.

## Appendix

Multimedia Appendix 1 CONSORT-eHEALTH Checklist (V 1.6.1).

Multimedia Appendix 2 Outline of intervention and standard care course format.

Multimedia Appendix 3 Participant flow diagram. IC: intervention course; MSOC: Multiple Sclerosis Online Course; SCC: standard care course.

Multimedia Appendix 4 One-month postcourse completion interview schedule.

## Abbreviations

APA: American Psychological Association
CONSORT-R: Consolidated Standards of Reporting Trials–Routine
COREQ: Consolidated Criteria for Reporting Qualitative Research
CSM: Common-Sense Model of Self-Regulation Theory
HADS: Hospital Anxiety and Depression Scale
IC: intervention course
IPQ: Illness Perceptions Questionnaire
MS: multiple sclerosis
MSOC: Multiple Sclerosis Online Course
MSSE: Multiple Sclerosis Self-Efficacy
PDDS: Patient-Determined Disease Steps
QoL: quality of life
RCT: randomized controlled trial
SCC: standard care course
WEIRD: Western, educated, industrialized, rich, and democratic

## Data Availability

The datasets generated during and/or analyzed during this study are available from the corresponding author on reasonable request.
