# Supplementary material for: Perceptions of Illness Control, Coherence, and Self-Efficacy Following a Web-Based Lifestyle Program for Multiple Sclerosis: A Qualitative Analysis of Semistructured Interviews
Source: J Med Internet Res. 2024 Nov 29;26:e60240. doi: 10.2196/60240 (PMC11645510; doi:10.2196/60240)
Supplement: Multimedia Appendix 2 [file jmir_v26i1e60240_app2.docx]

| Week | Modules | Intervention | Standard-care |
| --- | --- | --- | --- |
| 1 | **Introduction** | Introductions of course practitioners and tutorial on course functionalities, format and timing | |
|  | **Diet and supplementation** | A plant-based wholefood diet plus seafood, with very low saturated fat (<20g/day); No dairy, meat, palm, or coconut oil. Omega-3 fatty acid supplement use (20-40ml per day). | Eat a balanced diet and follow national guidelines. Common diets: gluten free, Paleolithic, McDougal, and Mediterranean diets. Alcohol consumption follow national guidelines |
| 2 | **Vitamin D and sunlight;** | Sunlight 15min/day 3-5 times/week; Vitamin D3 supplement use ≥5000IU/day | No specific recommendations. |
|  | **Physical activity** | 20-30 mins, 5 times/week physical activity | 30 mins moderate aerobic activity and strength training 2 times/week |
| 3 | **Stress reduction** | 30 min/day meditation | No conclusive evidence (no information provided) |
|  | **Family and prevention** | Genetic risk of getting MS and prevention via lifestyle; no smoking; role of medication | Genetic risk of getting MS, smoking increases MS; role of medication |
| 4 | **Concluding remarks** | Concluding remarks and closing ceremony | |
| 5/6 | **Catch-up** |  | |

**Multimedia Appendix 2.** Outline of intervention and standard care course format
