# Supplementary figures and images for: Perceptions of Illness Control, Coherence, and Self-Efficacy Following a Web-Based Lifestyle Program for Multiple Sclerosis: A Qualitative Analysis of Semistructured Interviews
Source: J Med Internet Res. 2024 Nov 29;26:e60240. doi: 10.2196/60240 (PMC11645510; doi:10.2196/60240)

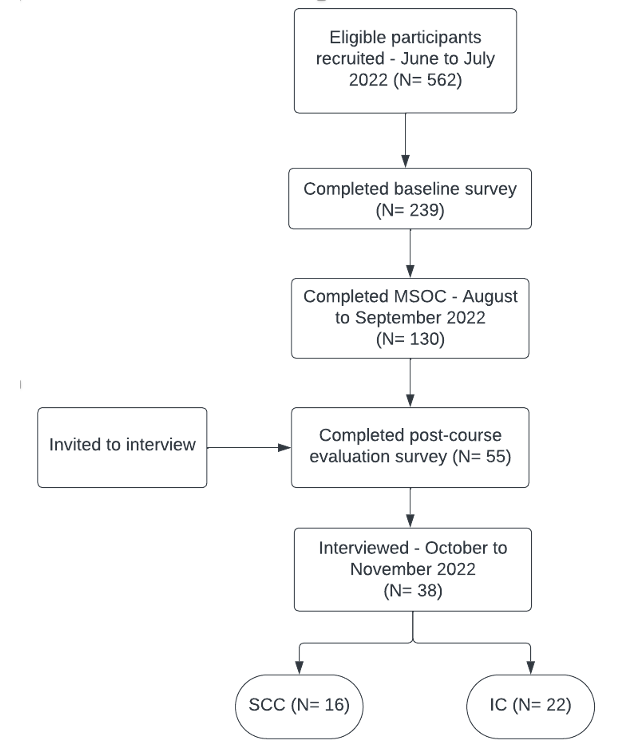

Supplement: Multimedia Appendix 3 [file jmir_v26i1e60240_app3.png]
